# Jan Beneken (1934–2021): European modeling and simulation pioneer

**DOI:** 10.1186/s41077-021-00195-9

**Published:** 2021-12-14

**Authors:** Willem L. van Meurs, Timothy A. J. Antonius

**Affiliations:** 1 Consultant in simulations, 11, Impasse des Balas, 65700 Lahitte-Toupière, France; 2 Department of Pediatrics, Division of Neonatology, Radboudumc Amalia Children’s Hospital, P.O. Box 9101, 6500 HB Nijmegen, The Netherlands

**Keywords:** Obituary, Modeling, Simulation

## Abstract

This obituary highlights a number of contributions by Professor Jan Beneken (1934–2021) to modeling of human physiology and pharmacology and to simulation-based training.

## Background

European modeling and simulation pioneer Professor Jan Beneken, born in Arnhem, The Netherlands on 20 March 1934, passed away in Eindhoven, The Netherlands, on 23 September 2021. This obituary focusses on the impact of Dr. Beneken on simulation-based training, established via his groundbreaking research on modeling of human physiology and pharmacology, via his mentorship of the team at the University of Florida that invented the Human Patient Simulator, and via the recent Jan Beneken conferences.

## Career overview

Dr. Beneken obtained his electrical engineering degree from the University of Delft, The Netherlands, in 1958, and his PhD in mathematics and natural sciences from the University of Utrecht, The Netherlands, in 1965. After working for the Netherlands Organization for Applied Scientific Research, he became a full-time professor of Medical Electrical Engineering at the Technical University, Eindhoven, The Netherlands, and served in that function from 1973 until 1995. He was the dean of the Faculty of Electrical Engineering from 1993 until 1995. He was the first president of the COMAC-BME, a coordinating committee of the European Commission for the research area Biomedical Engineering, from 1982 until 1990. He was a co-founder of the European Society for Engineering and Medicine (ESEM), and one of the founders of the journal Technology in Healthcare [1]. In 1995, Dr. Beneken was knighted in the order of the Dutch Lion for exceptional merits to society.

## Modeling of human physiology

His 1965 PhD thesis [2] presents a model that is still used in many simulators today and elaborated upon in many publications; see for example Goodwin et al. [3]. He met American physiologist Arthur Guyton in Leiden, The Netherlands, and was invited to work with him in Jackson, MS in 1965. His 1968 paper with Vincent Rideout, from the University of Wisconsin [4], allows for the coupling of cardiovascular and respiratory models, and his 1972 paper with Ty Smith and Aart Zwart [5] lays the basis for coupling cardiovascular models and models of anesthetic gas transport.

## Invention of the human patient simulator

Drs. Beneken and Joachim (Nik) Gravenstein acted as mentors to the team at the University of Florida that invented the Human Patient Simulator between 1988 and 1994 [6, 7]. Together, they demonstrated how an engineer and a medical specialist could cross the boundaries of their respective disciplines and come to highly effective technical solutions to medical problems. Dr. Beneken’s specific contributions included acting as a sounding board to the developers of the models of human physiology and pharmacology, which played a major role in making the Human Patient Simulator the most reactive simulator on the market. Dr. Beneken received the University of Florida Distinguished Achievement Award in 1995.

## The Jan Beneken conferences

**Fig. 1:**
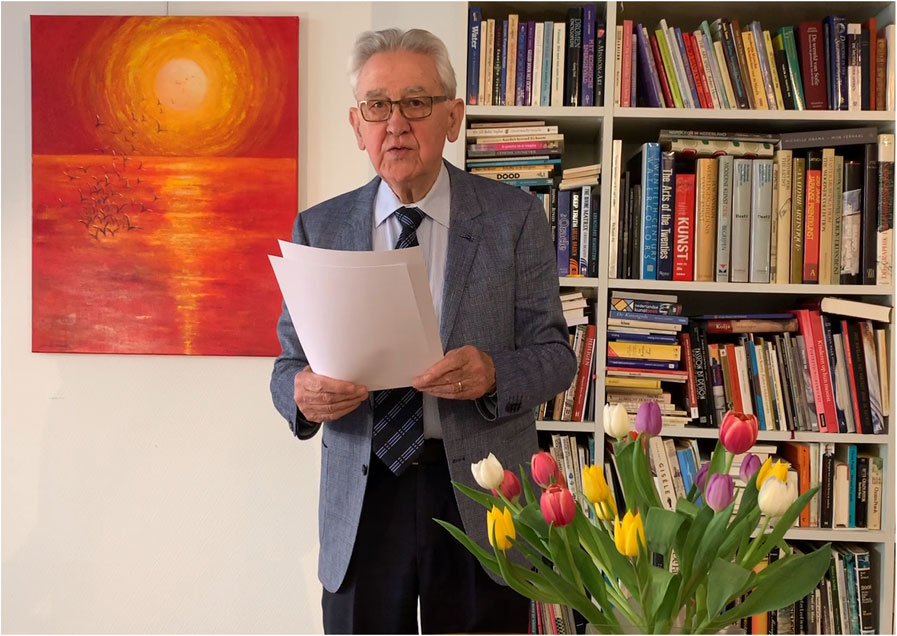
Dr. Beneken presenting at the 2019 AMAC conference. Reproduced with permission from the video by Dr. Berend Westerhof [9]. Painting in the background: *Vrijheid VII* © 2009 Gertrude Beneken-Erkelens

Two Jan Beneken conferences on modeling and simulation of human physiology took place in Eindhoven, The Netherlands, in 2013, and in Nijmegen, The Netherlands, in 2015. These conferences brought together several generations of Dutch and some other European modelers. Dr. Beneken was actively involved in the organization of the conferences and a stimulating presence during the debates. In 2017, the conference was held at the University of Twente, Enschede, The Netherlands, and with Dr. Beneken’s assent renamed to Applied Modeling in Acute Care (AMAC). The broader international coverage of the conferences was confirmed in 2019, when the conference was held in Canberra, Australia. The keynote lecture was given by Nobel prize winner Dr. Brian Schmidt, and Dr. Debra Nestel, Editor in Chief of Advances in Simulation at the time, presented a plenary lecture. Dr. Beneken pre-recorded a brief overview of his career and presented the Jan Beneken award to a promising young modeling researcher from the Australian National University [8] (Fig. 1).

## Conclusion

Via his research on modeling of human physiology and pharmacology, his mentorship of the team that invented the Human Patient Simulator, and his active participation in the conferences that bear his name, Professor Jan Beneken (1934-2021) made decisive contributions to simulation-based training in Europe and beyond.

## Data Availability

An email by Dr. Westerhof giving permission to use the picture is on file.

## Abbreviations

AMAC: Applied modeling in acute care
COMAC-BME: Coordinating committee of the European commission for the research area biomedical engineering
ESEM: European society for engineering and medicine
